# Generation of monocyte-derived tumor-associated macrophages using tumor-conditioned media provides a novel method to study tumor-associated macrophages in vitro

**DOI:** 10.1186/s40425-019-0622-0

**Published:** 2019-05-28

**Authors:** Brooke Benner, Luke Scarberry, Lorena P. Suarez-Kelly, Megan C. Duggan, Amanda R. Campbell, Emily Smith, Gabriella Lapurga, Kallie Jiang, Jonathan P. Butchar, Susheela Tridandapani, John Harrison Howard, Robert A. Baiocchi, Thomas A. Mace, William E. Carson

**Affiliations:** 1 Comprehensive Cancer Center, The Ohio State University, Columbus, OH USA; 2 Division of Surgical Oncology, The Ohio State University, N924 Doan Hall, 410 W. 10th Avenue, Columbus, OH 43210 USA; 3 Division of Hematology, Department of Medicine, The Ohio State University, Columbus, OH USA

**Keywords:** Tumor-associated macrophages, In vitro generation, Cancer, Immunotherapy

## Abstract

**Background:**

Tumor-associated macrophages (TAM) are expanded and exhibit tumor-promoting properties within the tumor microenvironment. Current methods to study TAM have not been replicated across cancer types and often do not include exogenous growth factors from the tumor, a key factor in TAM differentiation in vivo.

**Methods:**

In this study, an in vitro method to generate monocyte- derived TAM using tumor- conditioned media (TCM) and a cytokine cocktail containing IL-4, IL-10, and M-CSF was utilized to study the phenotype, morphology, and function of TAM across multiple cancer types. TCM was generated from two breast cancer cell lines and an Epstein-Barr virus-positive lymphoma cell line. The properties of in vitro generated TAM were compared to in vitro generated M1 and M2- like macrophages and TAM isolated from patients with cancer.

**Results:**

TAM generated in this fashion displayed an increase in CD163/CD206 co-expression compared to M2- like macrophages (87 and 36%, respectively). TAM generated in vitro exhibited increased transcript levels of the functional markers IL-6, IL-10, CCL2, c-Myc, iNOS, and arginase compared to in vitro generated M2-like macrophages. Functionally, in vitro generated TAM inhibited the proliferation of T cells (47% decrease from M1-like macrophages) and the production of IFN-γ by natural killer cells was inhibited (44%) when co-cultured with TAM. Furthermore, in vitro generated TAM secreted soluble factors that promote the growth and survival of tumor cells.

**Conclusions:**

Limited access to patient TAM highlights the need for methods to generate TAM in vitro. Our data confirm that monocyte-derived TAM can be generated reliably using TCM plus the cytokine cocktail of IL-4, IL-10, and M-CSF. Given the ability of TAM to inhibit immune cell function, continued study of methods to deplete or deactivate TAM in the setting of cancer are warranted.

**Electronic supplementary material:**

The online version of this article (10.1186/s40425-019-0622-0) contains supplementary material, which is available to authorized users.

## Introduction

Tumor progression is influenced by growth factors and cytokines released from an intricate network of immune cells in the tumor microenvironment that significantly contribute to tumor progression [1]. For example, myeloid-derived suppressor cells (MDSC), regulatory T cells, and tumor-associated macrophages (TAM) exist in the tumor microenvironment and have been found to promote tumor growth both directly and indirectly [2–4]. Clinically, the presence of TAM has been associated with a poor prognosis in cancer patients, likely due to their ability to enhance tumor cell proliferation, promote metastasis, and stimulate angiogenesis [5]. These factors highlight TAM as a potential therapeutic target in cancer and provide a rationale for exploring the relationship between TAM and malignant cells.

Broadly, macrophages exist in two major phenotypes, labeled as M1 and M2. Although this classification system can assist the researcher in many ways, it is also possible that this approach could lead to over-simplification of the properties of different macrophage populations. Indeed, the M1/M2 schema has seen the development of multiple sub-types. Classically activated M1 macrophages receive stimulation from IFN-γ, granulocyte-macrophage colony-stimulating factor (GM-CSF), and LPS to produce IL-12 and IL-23 and are involved in promoting type 1 T helper cell (Th1) responses to infection [6]. In contrast, M2 macrophages are alternatively activated by cytokines such as macrophage colony-stimulating factor (M-CSF), IL- 4, IL-10, and IL-13 and produce anti-inflammatory cytokines such as IL-10 and TGF-β. M2 macrophages are able to limit tissue damage caused by inflammatory processes and stimulate tissue repair and remodeling. In vitro, M1-like macrophages can be differentiated from monocytes by stimulation with GM-CSF, LPS, and IFN-γ while M2-like macrophages can be differentiated from monocytes using M-CSF and IL-4. TAM are widely considered to be M2-like macrophages in that they can support tumor growth and suppress local immunity. However, recent studies have concluded that TAM are influenced by environmental factors, such as location within the tumor microenvironment and tumor-derived factors [7–9]. Macrophages have high plasticity and respond to their microenvironment. Therefore, we hypothesize that TAM can be generated in vitro using known methods for differentiating monocytes to M2-like macrophages with the addition of tumor- conditioned media from the tumor type of interest.

Monocytes can be functionally reprogrammed to polarized phenotypes by exposure to exogenous growth factors [10]. In the setting of cancer, monocytes can be recruited from circulation and differentiated into macrophages by M-CSF. Once monocytes are recruited to the tumor, factors in the tumor microenvironment rapidly promote their differentiation into TAM. Tumor-derived factors like IL-10 and IL-4 inhibit the production of pro-inflammatory cytokines and chemokines in macrophages and create an immunosuppressive M2-like phenotype resembling TAM [11]. Therefore, these factors (M-CSF, IL-4, and IL-10) were utilized to generate monocyte-derived TAM in vitro. The tumor-derived factors chosen in this study are consistent with factors found in the tumor microenvironment of various malignancies and are modified from the M2 generation protocol published by Rey-Girand et al. This protocol was modified to fit our hypothesis that tumor-derived factors found in media conditioned from tumor cells (tumor-conditioned media) is needed to polarize monocytes to a TAM phenotype. Therefore, a recipe consisting of IL-4, IL-10, M-CSF, and tumor-conditioned media was utilized in this study to generate monocyte-derived TAM in vitro.

Access to patient TAM is limited. Therefore, in vitro differentiation protocols that recapitulate the phenotype and function of TAM are needed to test new therapeutic intervention strategies that specifically target TAM. However, previously published methods of culturing monocytes for TAM polarization have not been tested and compared across multiple cancer types. Here we have investigated a novel method to generate TAM in vitro that is reproducible in multiple types of cancer. Monocytes were differentiated and polarized into TAM using tumor-conditioned media (TCM) from breast cancer or lymphoma tumor cells and a cytokine cocktail consisting of IL-4, IL-10, and M-CSF. We evaluated the phenotype, morphology, and function of TAM generated via this method in vitro in comparison to in vitro classically activated (M1-like) and alternatively activated (M2-like) macrophages, and to TAM isolated from patient tumor samples.

## Materials and methods

### Cytokines

Cytokines employed were recombinant human IL-4 (Shenandoah, 100–09), recombinant human IL-10 (Peprotech, 200–10), recombinant human M-CSF (Shenandoah, 100–03), recombinant human GM-CSF (R&D Systems, 215-GM-010), recombinant human IL-6 (Shenandoah, 100–10), human IFN-γ recombinant protein (ThermoFisher Scientific, RIFNG100), and recombinant human IL-15 (Shenandoah, 100–86).

### Cell lines

The SK-BR-3 and MDA-MB 231 human breast adenocarcinoma cell lines were obtained from American Type Culture Collection. The LK 46 lymphoblastoid cell line (LCL) was generated by infecting naïve B cells with the B95.8 strain of Epstein-Barr virus (EBV). Upon establishment of EBV immortalized lymphoblasts, cells were subcloned and LCLs expanded. EBV gene product expression was validated by immunoblot and shown to be consistent with a type III latency profile. Cells were maintained in RPMI 1640 (SK-BR-3 and LK 46) or DMEM (MDA-MB 231) medium containing 10% fetal bovine serum (FBS) and penicillin/streptomycin.

### Harvesting tumor-conditioned media

To obtain culture supernatants for the generation of tumor-conditioned media, approximately 2 × 10^5^ cancer cells per 1 mL were grown to 80% confluence and incubated in 0.2% FBS medium for 24 h. After incubation, the TCM was harvested and centrifuged to remove suspended cells. The supernatant was collected and 10% FBS was added to reconstitute the medium.

### Isolation of human monocytes, natural killer (NK) cells, and T cells

Human monocytes, NK cells, and T cells were prepared from healthy donor blood (American Red Cross, Columbus, OH) samples by utilizing the Human RosetteSep Monocyte, NK cell, and T cell Enrichment Cocktail negative selections (STEMCELL Technologies, Vancouver, BC, Canada) respectively and according to the manufacturer’s instruction followed by a Ficoll hypaque density gradient centrifugation technique [12]. Briefly, the appropriate RosetteSep Enrichment Cocktail was added to whole blood at a concentration of 50 μL/mL of whole blood. After incubation at room temperature for 20 min, the blood sample was diluted with an equal volume of PBS and mixed gently. The sample was carefully layered on top of the Ficoll density gradient medium and centrifuged with the brake off. Enriched cell populations were removed from the density gradient medium (plasma interface). After washing with PBS, enriched cells were lysed with red blood cell lysis buffer to remove residual red blood cells. Purity greater than 94% for monocytes, NK cells, and T cells was confirmed via flow cytometric analysis according to CD14, CD56, and CD3 surface marker expression respectively. Monocytes were cultured in 10% HAB medium. NK cells were cultured in RPMI-1640 medium supplemented with 10 ng/mL IL-15. T cells were frozen in freezing media (FBS + 10% DMSO) until the generation of macrophages was complete.

### Generation of tumor-associated macrophages

Freshly isolated healthy donor human CD14^+^ monocytes were plated in a 100 × 20 mm cell culture dish (USA Scientific) at a concentration of 1 × 10^6^ cells per 1 mL media. Monocytes were cultured in a 1:1 ratio of 10% HAB medium and TCM plus the addition of IL-4 (1 μg/mL), IL-10 (1 μg/mL), and M-CSF (1 μg/mL). Medium and cytokines were refreshed every other day and cells were harvested on day 7 using a non-enzymatic cell dissociation solution (Sigma Aldrich).

### Monocyte differentiation to M1/M2-like macrophages

For M1/M2 monocyte differentiation freshly isolated human CD14^+^ monocytes were plated in a 100 × 20 mm tissue culture dish (USA Scientific) at a concentration of 1 × 10^6^ cells per 1 mL of 10% HAB medium. For M1-like monocyte differentiation GM-CSF (1 μg/mL) was added to fresh media every other day. On day 6, media was refreshed with GM-CSF (1 μg/mL), IFN-γ (20 ng/mL), and LPS (50 ng/mL) for 24 h. For M2-like monocyte differentiation M-CSF (1 μg/mL) was added to fresh media every other day. On day 6, media was refreshed with M-CSF (1 μg/mL) and IL-4 (1 μg/mL) for 24 h. Both differentiation conditions were harvested on day 7 using a non-enzymatic cell dissociation solution (Sigma Aldrich). This protocol is based on the Rey-Giraud et al. M1/M2 macrophage generation protocol.

### Flow cytometry

Antibodies used for cell labeling of isolated human monocytes, NK cells, T cells, in vitro generated TAM, M1-like, or M2-like macrophages, or single cell suspensions isolated from tumor or normal human tissue samples were as follows: V450 anti-CD14 (BD Biosciences, 560,349), PE anti-CD56 (Beckman Coulter, IM2073U), PE anti-CD3 (Beckman Coulter, IM1282), PE anti-CD19 (BD Biosciences, 555,413), PE-CF594 anti-CD163 (BD Biosciences, 562,670), PE/Cy7 anti-CD45 (BioLegend, 304,016), FITC anti-CX3CR1 (BioLegend, 341,605), PE anti-CD163 (BioLegend, 333,605), APC anti-CD206 (BD Biosciences, 561,783), and PerCP/Cy5.5 anti-PD-L1 (BioLegend, 329,737). Flow cytometry was performed on a LSR II flow cytometer.

### Real-time PCR

Total RNA was extracted using TRizol reagent (Life Technologies). Reverse transcription reactions were performed using 500 ng RNA in a 20 μL reaction with the high-capacity reverse transcription kit (Life Technologies). cDNA was used as a template to measure the expression of human CD206, PD-L1, IL-6, IL-10, CCL2, c-Myc, MMP9, CSF-1R, iNOS, arginase, TNF-α and VEGF genes by quantitative real-time PCR. Human β-actin served as an internal control for each reaction (Life Technologies). Real-time PCR reactions were performed using the ABI PRISM 7900HT fast Real-time PCR system with SYBER Green (Applied Biosystems) and TaqMan (ThermoFisher Scientific) chemistry.

### Human tissue processing

Tumor and normal tissues collected from patients with metastatic melanoma were cut into 1–2 mm^3^ sections and transferred to a GentleMACS C tube containing PBS. The C tube was run on the GentleMACS dissociator. Dissociated cells were passed through a 70 μm cell strainer. Cells were washed with PBS and stained for flow cytometry. Samples were acquired after patient informed consent was obtained under an Institutional Review Board (IRB)-approved protocol for human subject research (IRB protocol 1999C0348).

### T cell CFSE assay

M1/M2-like macrophages and MDA-MB 231 conditioned TAM were generated from healthy donor monocytes as described in Materials and Methods. Autologous T cells were isolated from healthy donor blood. T cells were labeled with CFSE (Life Technologies), non-specifically activated with anti-CD3/CD28 beads (Life Technologies), and co-cultured at a 1:1 ratio. After three days T cell proliferation was assessed by flow cytometry.

### NK cell co-culture

Monocytes and autologous NK cells were procured from a healthy donor leukopack and M1/M2- like macrophages and MDA-MB 231 conditioned TAM were generated in vitro. NK cells were added to 96 wells coated with polyclonal human IgG and stimulated with IL-12 (20 ng/mL) and IL-18 (50 ng/mL). Immune cells were added at a 1:1 ratio. M1, M2, and TAM cultured alone in the presence of stimulus were utilized as negative controls. After three days of incubation, supernatants were harvested and analyzed for human IFN-γ using a Duoset ELISA (R&D Systems, DY285).

### Measurement of supernatant cytokines and chemokines

For quantitative detection of cytokines and chemokines in macrophage and tumor-conditioned media supernatants, an unbiased pairwise screening of 15 antibodies was performed on U-PLEX plates using an electrochemilluminescence method and read on the Meso QuickPlex SQ 120 (Meso Scale Discovery, 1601 Research Boulevard, Rockville, MD [13]). All samples were run in batches to minimize inter-assay variability, assayed in duplicate, and quantitated using a standard curve.

### Statistical analysis

Statistical significance of differences between groups was analyzed by ANOVA and a two-tailed Student’s t test, and *P* ≤ 0.05 was considered to be statistically significant.

## Results

### Generation of tumor-conditioned media and in vitro macrophages

**Fig. 1:**
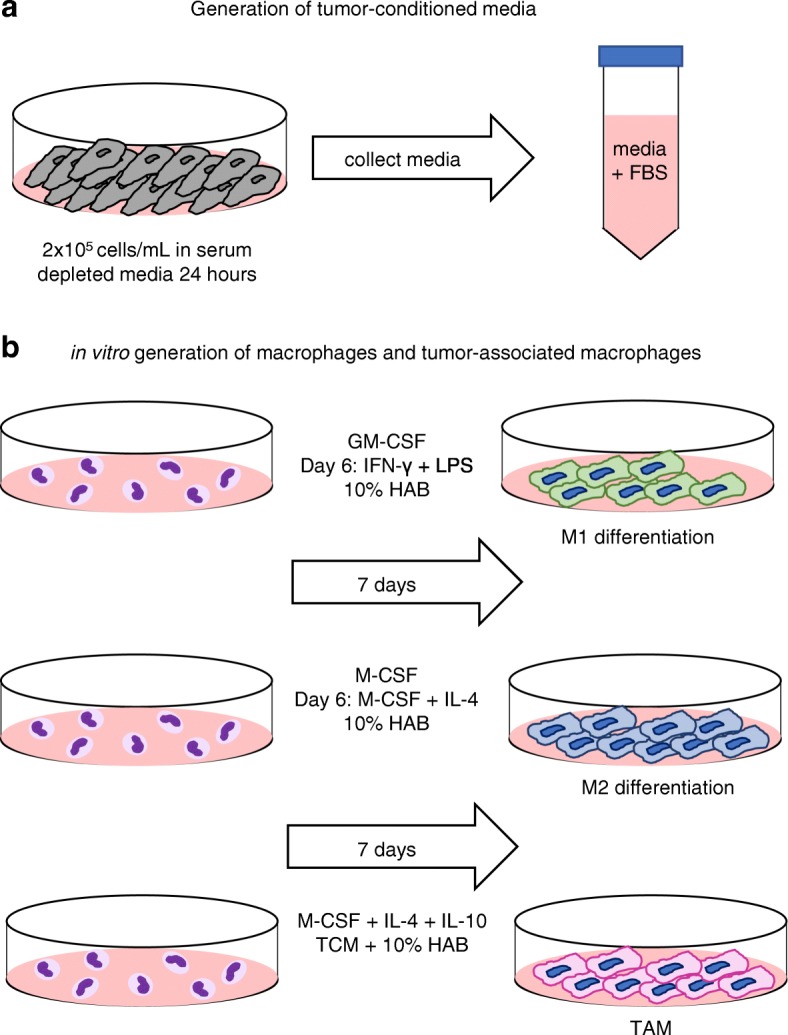
Generation of tumor-conditioned media and in vitro macrophages. (**a**) Schematic of the generation of tumor-conditioned media (TCM) using 2 × 10^5^ cells/mL (MDA- MB 231, SK-BR-3 or LK 46) in 0.2% FBS media for 24 h. Media was collected and centrifuged to remove any tumor cells. 10% FBS was added. TCM was stored in 5 mL aliquots in − 80° freezer until use. (**b**) Schematic of the in vitro generation of macrophages. Monocytes harvested from healthy donor blood were plated at 1 × 10^6^ cells/mL in a total of 10 mL 10% HAB (M1/M2) or 5 mL 10% HAB plus 5 mL TCM (TAM). In M1-like monocyte differentiation, GM-CSF (1 μg/mL) was added to culture for 6 days. On day 6 the media was refreshed with GM-CSF (1 μg/mL) + IFN-γ (20 ng/mL) + LPS (50 ng/mL) for 24 h. In M2-like differentiation, M-CSF (1 μg/mL) was added to culture for 6 days. On day 6 the media was refreshed with M-CSF (1 μg/mL) and IL-4 (1 μg/mL). In TAM differentiation, M-CSF, IL-10, and IL-4 (all at 1 μg/mL) were added to culture in combination with TCM and 10% HAB (1:1). In all differentiation protocols media was refreshed every other day and cells were harvested on day 7

The development of the macrophage phenotype is highly dependent upon cues from the tumor microenvironment. Therefore, media was generated containing soluble factors (Additional file 1: Figure S1) produced by tumor cells (tumor-conditioned media - TCM) and used to provide stimuli to monocytes in the context of an in vitro protocol for generating TAM. Specifically, TCM contained soluble factors responsible for the recruitment and adhesion of myeloid cells to the tumor including macrophage inflammatory protein-1β (MIP-1β), vascular endothelial growth factor-α (VEGF-α), IL-8, IFN- γ, and granulocyte colony-stimulating factor (G-CSF) [14, 15]. Two human breast cancer cell lines, MDA-MB 231 and SK-BR-3, and a human lymphoma cell line, LK 46, were tested in this system. Tumor cells were plated at 2 × 10^5^ cells/mL in a 24-well plate and allowed to adhere before the media was replaced with serum depleted media (0.2% FBS) for 24 h. Next, TCM was harvested, centrifuged to remove tumor cells, and the supernatant was restored to 10% FBS, resulting in TCM (Fig. 1A). Monocytes were isolated fresh from whole, healthy blood as described in Materials and Methods. M1-like macrophages were generated by culturing monocytes with GM-CSF in the presence of IFN-γ and LPS. M2-like macrophages were generated by culturing monocytes with M-CSF in the presence of IL-4. TAM were generated using monocytes cultured with TCM and a cytokine cocktail containing IL-4, IL-10, and M-CSF. In all polarization methods, the media was refreshed every other day and cells were harvested on day 7 (Fig. 1B).

### Characterization of in vitro generation methods of tumor-associated macrophages

**Fig. 2:**
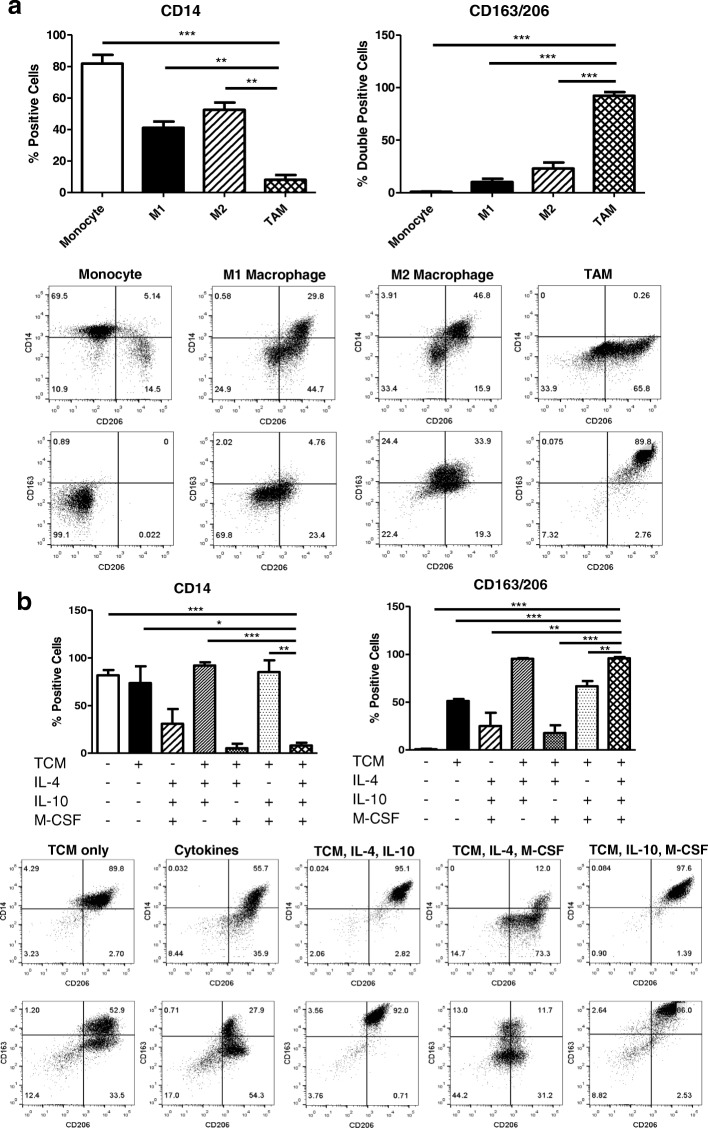
Characterization of in vitro generation methods of tumor-associated macrophages. (**a**) In vitro generated TAM were compared to classically and alternatively activated macrophages. Cells were stained on generation day 7 to assess surface expression of CD14, CD163, and CD206 analyzed by flow cytometry. Representative flow plots are shown. (**b**) The method used to generate TAM in vitro was validated using 6 conditions (TCM only, cytokines only, TCM + IL-4 + IL-10, TCM + IL-4 + M-CSF, TCM + IL-10 + M-CSF, TCM + IL- 4 + IL- 10 + M-CSF). Surface expression of CD14, CD163, and CD206 was measured on day 7 and analyzed by flow cytometry. Representative flow plots are shown

Franklin et al. reported that TAM are phenotypically different from traditional M2 macrophages in a mouse mammary cancer model [16]. This finding suggests that TAM could be the result of a tumor-elicited inflammatory response. Enriched CD14^+^ monocytes were cultured as described in Fig. 1B to generate M1-like macrophages, M2-like macrophages, and in vitro TAM. Cells were harvested on day 7 and flow stained for CD14, which is strongly expressed on the surface of monocytes. Indeed, in vitro generated TAM were phenotypically different from M1/M2 macrophages. As previously described, M1-like macrophages expressed significantly less CD14 compared to M2-like macrophages [6]. In addition, TAM expressed significantly less CD14 than both M1 and M2- like macrophages *(*Fig. 2A*)*. Cells were also stained for surface expression of CD163 (scavenger receptor) and CD206 (mannose receptor). TAM generated in vitro were identified by the co-expression of CD163 and CD206 [17]. TAM populations displayed significantly more expression of CD163/206 co-positive cells compared to M1 and M2-like macrophages (Fig. 2A).

Next, the in vitro TAM generation method was further characterized. Freshly isolated human monocytes were cultured in six different conditions. Tumor-conditioned media (TCM) only, cytokine only (IL-4, IL-10, and M-CSF), TCM with IL-4 and IL-10, TCM with IL-4 and M-CSF, TCM with IL-10 and M-CSF, and TCM with IL-4, IL-10, and M-CSF (the last condition being the in vitro TAM generation method). Cells were harvested on day 7 and assessed for surface expression of CD14, CD163, and CD206 (Fig. 2B). Although monocytes cultured with TCM, IL-4, and IL-10 expressed high levels of CD163/CD206 co-positive cells, the surface expression of CD14 was comparable to the monocyte control suggesting the phenotype of cells developed under this condition is different than TAM. (Fig. 2B). In conclusion, the differentiation recipe consisting of TCM with the cytokine cocktail (IL-4, IL-10, and M-CSF) is necessary to fully polarize monocytes to a TAM-like phenotype.

### Phenotype and functional characterization of in vitro generated TAM

**Fig. 3:**
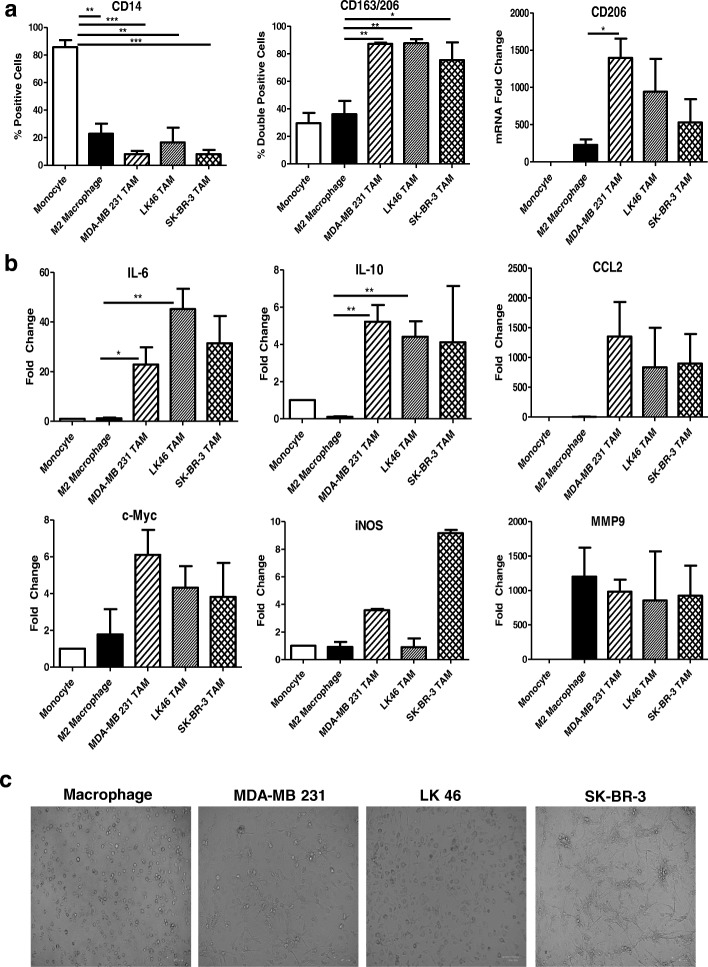
Phenotype and functional characterization of in vitro generated TAM. (**a**) M2-like macrophages and in vitro generated TAM were flow stained for surface expression of CD14 and CD163/CD206 co-expression and were analyzed by real-time PCR for transcript expression of CD206. (**b**) M2-like macrophages and in vitro generated TAM were analyzed by real-time PCR for transcript expression of IL-6 (macrophage-associated inflammatory pathway), IL-10 (immunosuppressive cytokine), CCL2 (macrophage stimulating chemokine), c-Myc (pro-tumor oncogene), iNOS (inducible nitric oxide synthase; tumor-protecting [54]), and MMP9 (matrix metallopeptidase 9; mediated extracellular matrix degradation and release of bioactive VEGFα [55]). (**c**) Brightfield images of in vitro generated macrophages and TAM generated using TCM from different tumor types at day 7

Initial results were shown in TAM generated using TCM from the triple negative human breast cancer cell line MDA-MB 231. In order to demonstrate the reproducibility of this method, the HER2-positive human breast cancer cell line, SK-BR-3, and a human lymphoma cell line, LK 46, were utilized in this system. Cells were harvested on day 7 of generation and assessed for surface expression of CD14 and CD163/CD206 co-positivity. M2-like macrophages were generated as a control. TAM generated from all three cell lines had significantly reduced CD14 surface expression compared to monocytes (Fig. 3A). This result demonstrates that TAM have differentiated from their monocytic origin. Additionally, TAM generated with TCM from all three cell lines had significantly increased co-positivity for CD163/CD206 compared to monocytes and in vitro generated M2-like macrophages (Fig. 3A). This result confirms that the method of polarizing monocytes with TCM and the cytokines IL-4, IL-10, and M-CSF is reproducible in multiple tumor types. In addition, mRNA expression of CD206 (M2-like macrophage marker) increased in TAM compared to M2-like macrophage controls when assessed by qRT-PCR (Fig. 3A).

To expand on these results, qRT-PCR was utilized to characterize the effector functions of in vitro generated TAM. Gene expression of IL-6, IL-10, CCL2, c-Myc, iNOS, MMP9, CSF-1R, arginase, TNF-α, and VEGF were evaluated (Fig. 3B and Additional file 1: Figure S1). Gene expression of IL- 6, IL-10, CCL2, c-Myc, iNOS, CSF-1R, and MMP9 was elevated in all three of the in vitro TAM preparations which suggests that in vitro generated TAM are immunosuppressive and thus tumor promoting. However, an increase in TNF- α and VEGF by qRT-PCR was not observed in these TAM (Additional file 1: Figure S2). This is a limitation of this system, but could be explained by a lack of a hypoxic environment. Tumor hypoxia is thought to be important in the activation of TAM and promotes the release of TNF-α and VEGF [18]. Consistent with previous results, TAM generated from MDA-MB 231, SK-BR-3, and LK46 TCM and the cytokines IL-4, IL-10, and M-CSF were morphologically different from M2- like in vitro generated macrophages. Macrophages were round and had a smooth surface, lacking cellular projections, while TAM were more spindle shaped with distinctly elongated cell projections (Fig. 3C).

### Phenotype of patient-derived TAM

**Fig. 4:**
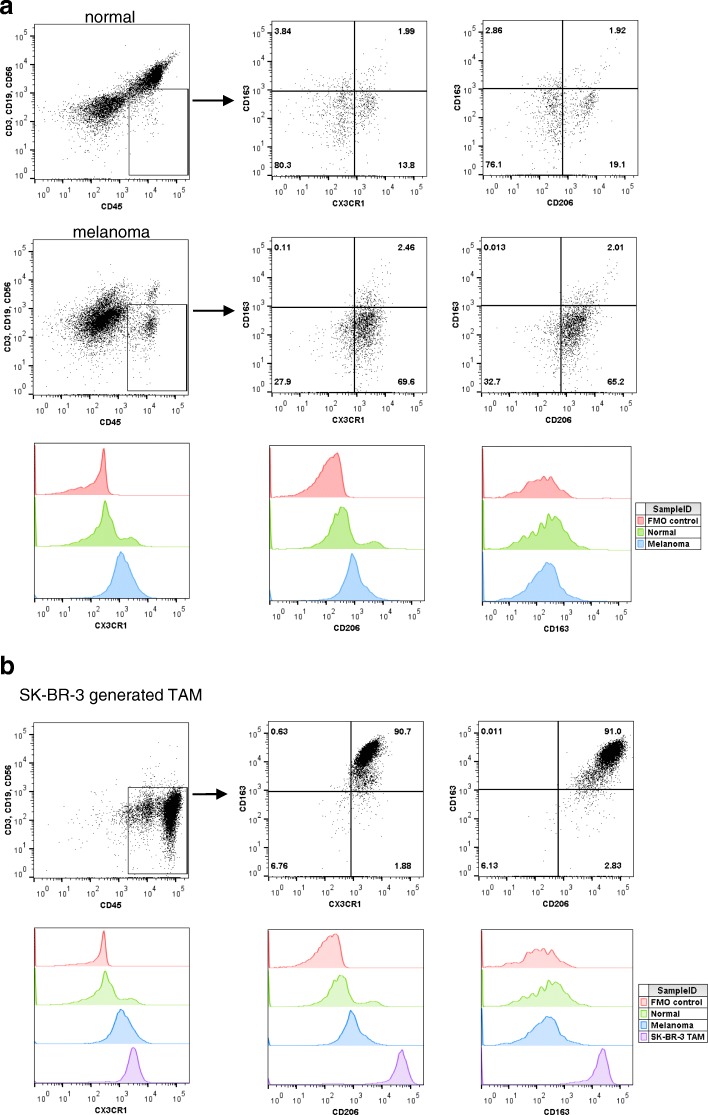
Human-derived TAM phenotype. (**a**) Melanoma and normal adjacent tissue were digested, stained for CX3CR1, CD163, and CD206 and analyzed by flow cytometry. Cell populations were first gated on the CD45^+^ and lineage^−^ (CD3, CD19, CD56) population. (**b**) SK-BR-3 in vitro generated TAMs were stained with antibodies for CX3CR1, CD163, and CD206 and analyzed by flow cytometry. Cell populations were first gated on the CD45^+^ and lineage^−^ (CD3, CD19, CD56) population

We next sought to compare in vitro generated TAM to TAM derived from the tumors of patients with advanced melanoma. Melanoma tumor and normal adjacent tissues were homogenized and processed according to the schema outlined in Materials and Methods. The single cell suspensions were stained for CD3, CD19, CD56, CD45, CD163, CD206, and CX3CR1. TAM are defined as lineage negative (CD3^−^, CD19^−^, CD56^−^) and CD45^+^. In addition, human TAM are considered to be positive for CD163, CD206, and CX3CR1 (fractalkine receptor or G-protein coupled receptor 13) [19]. Macrophages have been shown to represent roughly 50% of tumor-resident CD45^+^ cells in some malignancies [16]. Therefore, cells were first gated cells positive for CD45 and lineage negative to identify a population of TAM origin. Cells were then gated for expression of CD163, CD206, or CX3CR1 [17, 20, 21]. Cells from melanoma tumor tissue were first compared to those from normal adjacent tissue. While the population of cells representing the TAM phenotype was relatively low in both tissues, a larger population was found in the melanoma tumor tissue compared to the normal tissue (Fig. 4A). This result is consistent with the idea that TAM can expand following their differentiation in tumors [22]. Next, in vitro generated TAM from SK- BR- 3 TCM and the cytokines IL-4, IL-10, and M-CSF were compared to TAM derived from melanoma tumor tissue using the same flow panel and gating strategy. In vitro generated TAM contained a population similar to the melanoma tumor tissue yet was a purer population, which is to be expected (Fig. 4B). In conclusion, in vitro generated TAM show a similar phenotype to TAM found in patient tumors and thus are a candidate for utilization as a tool to study TAM in vitro.

### In vitro generated TAM inhibit T cell and NK cell function

**Fig. 5:**
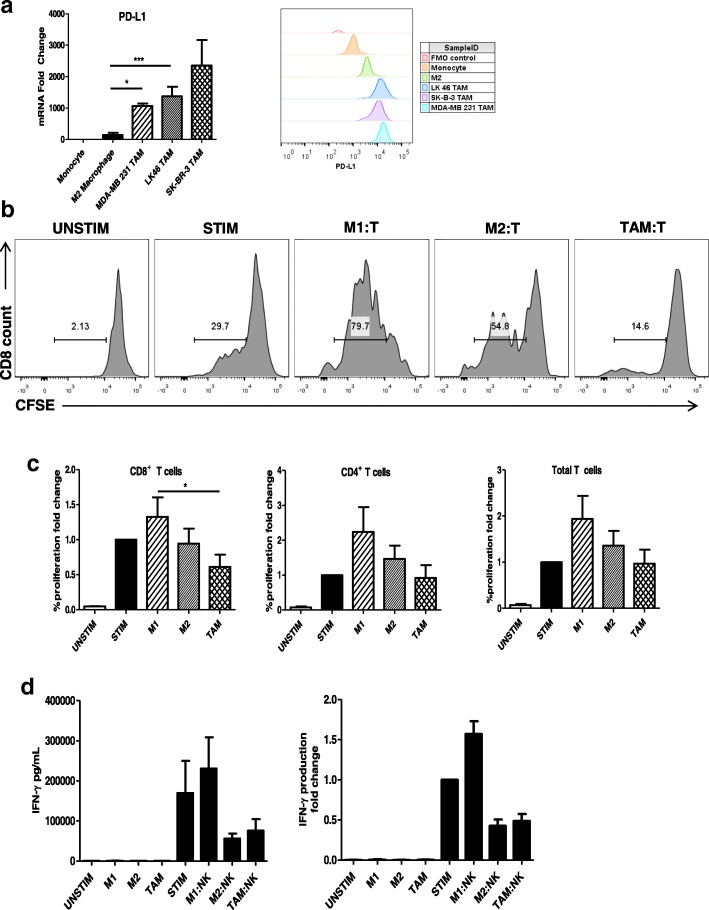
in vitro generated TAM inhibit T cell and NK cell function. (**a**) M2-like macrophages and in vitro generated TAM were analyzed by real-time PCR for transcript expression and flow cytometry for surface expression of PD-L1 CD3/CD28 bead activated T cells labelled with CFSE were co-cultured with M1-like and M2-like macrophages and MDA-MB 231 conditioned in vitro generated TAM at a 1:1 ratio. After 72 h cells were collected and (**b**) proliferation was evaluated by flow cytometry. Histograms show results from one representative experiment of CD8^+^ T cells. (**c**) Percent T cell proliferation expressed as a fold change and percentage. Bars represent the mean concentration ± SD of T cell proliferation analyzed by flow cytometry. Data are representative of three independent experiments with similar results; *p* < 0.05. (**d**) NK cells stimulated with IL-12, IL-18 and immobilized IgG were co-cultured with M1-like and M2- like macrophages and MDA-MB 231-conditioned in vitro generated TAM at a 1:1 ratio for 72 h. Bars represent the mean concentration ± SD of IFN-γ content from culture supernatants analyzed by ELISA. Data are representative of three independent experiments with similar results

The functional characterization of TAM remains a major area of investigation. Recent reports have demonstrated that TAM have the ability to enhance tumor cell proliferation, invasion, metastasis, and stimulation of angiogenesis [2]. Notably, qRT-PCR and flow cytometry analysis revealed the expression of programmed cell death-ligand 1 (PD-L1) on in vitro generated TAM (Fig. 5A). One mechanism by which TAM promote tumor generation is through their ability to inhibit T cell proliferation. Given the hypothesis that TAM regulate T cell suppression through the expression of PD-L1 we evaluated the ability of in vitro generated TAM to suppress T cell proliferation [23, 24]. T cells were labeled with CFSE and activated with anti-CD3/CD28 beads and cultured with M1 and M2-like macrophages and in vitro generated TAM at a ratio of 1:1. T cells co-cultured with M1-like macrophages exhibited enhanced proliferation, while T cells co- cultured with M2-like and in vitro generated TAM displayed reduced proliferation (Fig. 5B and C).

**Fig. 6:**
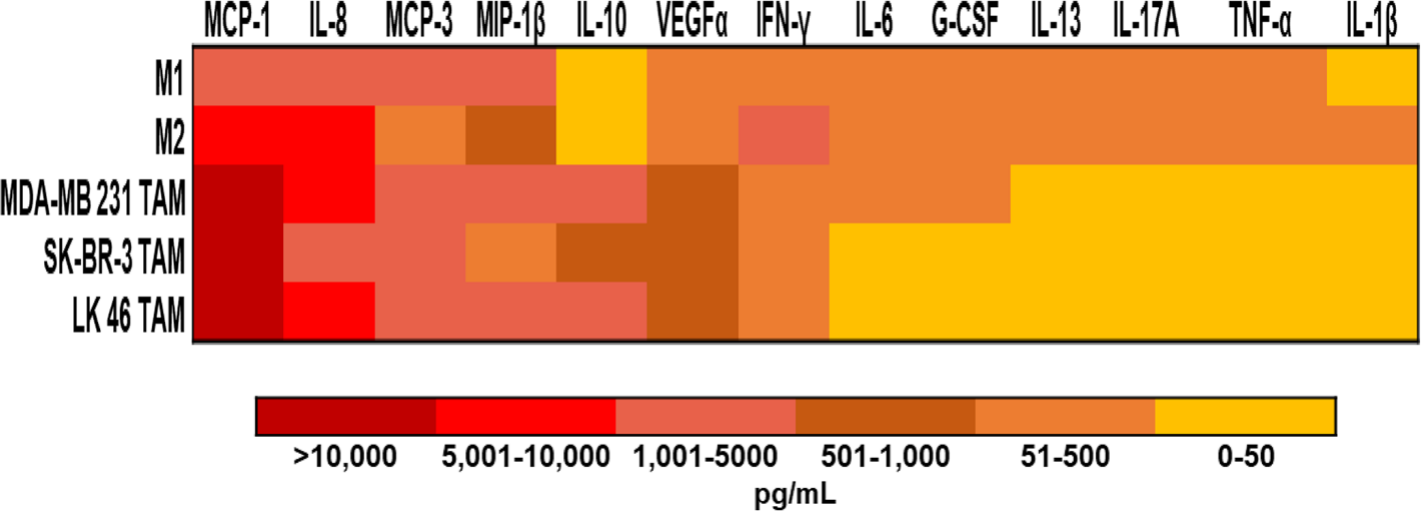
in vitro generated TAM secrete growth factors that support the growth and survival of tumor cells. In vitro generated TAM secrete inflammatory cytokines. Culture supernatants were harvested from M1 and M2-like macrophages and in vitro generated TAM conditioned from MDA-MB 231, SK- BR-3, and LK 46 tumor-conditioned media. Data are presented as a heat map for expression of soluble factors in supernatants from each macrophage phenotype

We and others have shown that myeloid cells can impair NK cell expression of IFN-γ and thus impact NK cell function, potentially impairing tumor surveillance [25]. In addition, we have previously reported that normal monocytes can enhance the NK cell cytokine response due to an interaction between the NKG2D activating receptor on NK cells and its ligand MICA on monocytes [26]. Therefore, we investigated the impact of different monocyte-derived macrophage phenotypes, including TAM, on the NK cell IFN-γ response to stimulation. To investigate the effect of immune cells on NK cell IFN-γ production, NK cells stimulated with plate bound IgG, IL- 12, and IL-18 were co-cultured alone, with M1- like macrophages, M2-like macrophages, or in vitro generated TAM at a ratio of 1:1. Indeed, an enhanced NK cell cytokine response was observed when NK cells were co-cultured with M1-like macrophages [27]. However, when NK cells were co-cultured with M2-like macrophages or in vitro generated TAM, NK cell production of IFN-γ was inhibited (Fig. 5D).

### In vitro generated TAM secrete growth factors

In vitro generated TAM produce inflammatory cytokines and growth factors that support the survival of tumor cells. We therefore wished to perform a comprehensive analysis of the immunomodulatory factors derived from the TAM being studied in this report. Supernatants from M1 and M2-like macrophages and in vitro generated TAM conditioned with MDA-MB 231, SK- BR-3, and LK 46 TCM were assessed using a U-PLEX -platform designed to assess the profile of soluble factors involved in the pro-tumor function of TAM. Overall, in vitro generated TAM had high expression of several tumor-derived chemoattractants including chemokines CCL2(MCP-1), CCL7(MCP-3), macrophage inflammatory protein (MIP)-1β, and vascular endothelial growth factor (VEGF)-α, which have been shown to promote monocyte recruitment to tumors in experimental mouse models of lung, breast, and pancreatic cancer [30–33] (Fig. 6).

## Discussion

The current study provides evidence of an effective method to generate and study TAM in vitro using TCM and the cytokines IL-4, IL-10, and M-CSF. The results demonstrate that in vitro generated TAM are phenotypically distinct from both M1 and M2- like macrophages and co- express the TAM markers CD163 and CD206. In vitro generated TAM share this phenotype with human TAM isolated from melanoma tumors. Interestingly, our study demonstrated crosstalk between TAM and NK cells and TAM and T cells. NK cells are lymphoid cells that are known to play a key role in cancer immunosurveillance. NK cell function was impaired when NK cells were co-cultured with TAM, an interaction that was characterized by a decrease in NK cell IFN-γ production. These results support the ability of TAM to impair the anti-tumor response of NK cells. While the suppressive factors produced by TAM that contribute to the inhibition of NK cells is not fully understood, it has been reported that expression of PD-L1 (highly expressed on in vitro generated TAM, Fig. 5A) and secretion of TGF-β by TAM are likely involved [23, 25, 28, 29].

In vitro generated TAM also impaired T cell function. The ability of in vitro generated TAM to inhibit CD8^+^ and CD4^+^ T cell proliferation suggests that targeting TAM in the setting of cancer could enhance NK and T cell function for improved cancer control. These results support the concept that in vitro generated TAM have the ability to impair the anti- tumor response of NK cells and T cells and suggests that PD- 1/PD-L1 directed therapies could exert a direct effect on macrophages in cancer types with implications for cancer treatment with checkpoint inhibitors.

Although protocols for in vitro differentiation of macrophages have been published, a detailed characterization of TAM generated in cultured tumor conditions has not yet been described. Several groups have reported protocols for generating M1/M2 macrophages that employ stimulation with GM-CSF and/or M-CSF, respectively [34–36]. Rey-Giraud et al. has described the influence of culture conditions on monocyte-derived macrophage phenotype and function. Several groups have reported on monocyte polarization methods that further subdivide M2 macrophages into M2a (cultured with M-CSF + IL-4 or IL-13) and M2c (cultured with M- CSF + IL-10) [37, 38]. This alternative M2 phenotype and function is similar to TAM. Extending these findings, we utilized a culture method that included TCM (media containing factors secreted from tumor cells) in combination with a cytokine cocktail derived from M2a and M2c macrophages including IL-4, IL-10, and M- CSF to develop a method of culturing TAM in vitro specific to the setting of cancer. Characterization of the TCM revealed the presence of several factors (MIP-1β, IFN-γ, and G-CSF) that could explain the phenotype of the in vitro generated TAM. Importantly, this method of TAM generation was reproduced using TCM from three different cell lines, suggesting that this method can be translated across cancer types.

TAM generated in vitro demonstrate an alternative phenotype that correlates with previously published data on M2 macrophages [39–41]. The ability of M-CSF to polarize monocytes to a M2-like phenotype is well documented, however TAM are considered a unique M2-skewed myeloid population which was confirmed in this study [8]. Higher expression of markers for immunosuppression, angiogenesis, and invasion were seen in TAM generated in vitro with TCM compared to M1 and M2-like macrophages. Most notably MCP-1, IL-10, and IL-8 were seen expressed in higher levels by TAM. MCP-1 is one of the most studied factors secreted by tumor cells and macrophages. MCP-1 has been shown to correlate with the number of TAM and with poor prognosis in human and murine tumors [42–44]. In human breast cancer models using MDA- MB 231, MCP-1 recruits monocytes to the tumor, driving growth and metastasis to the lung and bone in nude mice [45, 46]. In vitro generated TAM also produced IL-10, a potent immunosuppressive cytokine that could contribute to T cell regulation [47, 48]. Finally, the proinflammatory cytokine IL-8 has been shown to enhance invasive activity of tumor cells in a human colon cancer cell line [49]. In contrast, the cytokines IFN-γ, IL-6, G-CSF, IL- 13, IL-17A, TNF-α, and IL-1β were expressed at low levels in TAM. Notably, M1-like macrophages expressed the M1 macrophage produced cytokine IL-6, while M2-like macrophages expressed IL-13 [50, 51].

The communication pathways between tumor cells and TAM are intricate and modulation of a single signaling axis to target the immunosuppressive function of TAM may not be effective. The development of future therapeutic strategies to target TAM requires the identification of multiple driving forces in macrophage polarization from the tumor microenvironment. A more comprehensive analysis of TAM in different cancers, cancer stages, and tissue types could provide the basis for a more refined approach to developing TAM targeting strategies. Although this study is limited to the model of two breast cancer and one lymphoma cell line, the system developed in this study can be used with TCM from different cancer types as a first step in this process.

**Fig. 7:**
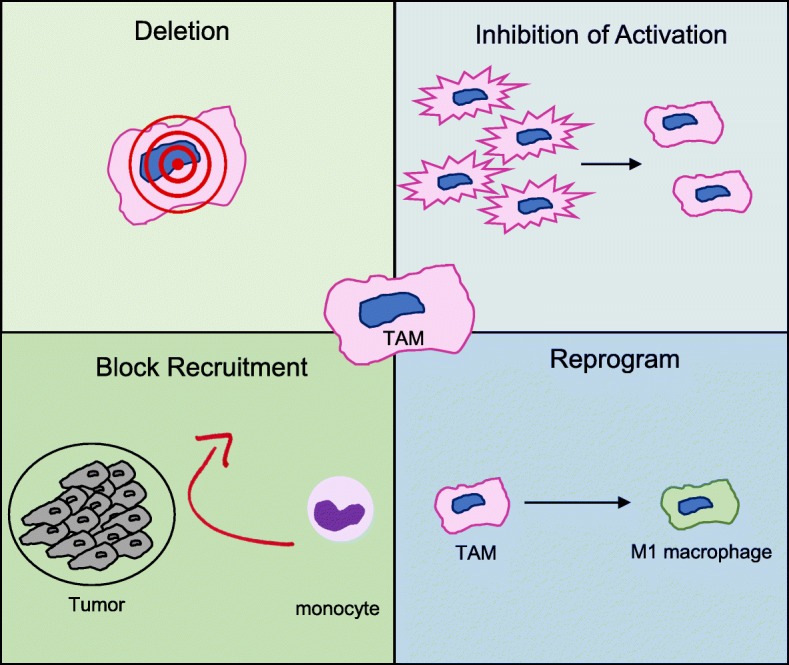
Therapeutic targeting of tumor-associated macrophages in cancer. TAM are a promising target for the immunotherapy of cancer. Several approaches to reduce TAM or functionally modify TAM include blocking monocyte recruitment to the tumor, deletion through antigen-specific targeting, reprogramming of TAM to M1 macrophages and inhibiting the activation of TAM

The role of TAM in promoting cancer has made TAM a novel target for the treatment of cancer. One of the most efficient strategies to target TAM to date is through the blockade of colony- stimulating factor-1 receptor (CSF-1R), which is known to be essential to the survival of TAM [52]. The CSF-1R antagonist PLX397 inhibits the infiltration of TAM into pancreatic tumors and reprograms remaining TAM to be less immunosuppressive in mice [53]. Furthermore, when PLX397 was combined with anti-PD-1 and anti-CTLA4 antibodies tumor expansion was completely blocked and even regressed compared to a 50% limit in tumor growth with checkpoint blockade alone compared to vehicle control. These results suggest that targeting TAM could boost the efficacy of checkpoint inhibitors. Interestingly, in vitro generated TAM express PD-L1 suggesting that PD-1/PD-L1 directed therapies could exert a direct effect on TAM. Other therapeutic strategies of targeting TAM include blocking monocyte recruitment to the tumor, reprogramming TAM to M1-like macrophages, and deletion of TAM through antigen-specific targeting (Fig. 7).

## Conclusions

In summary, TAM are involved in the progression of many human cancers. Therefore, it is important to gain a better understanding of how TAM contribute to cancer cell growth and to apply these findings clinically. Recent and ongoing studies targeting TAM demonstrate its promise as a potential cancer therapy. Our data support that in vitro generation of TAM using TCM and the cytokines IL-4, IL-10, and M-CSF provides a novel method to study the phenotype, morphology, and function of human TAM.

## Additional file

Additional file 1: **Figure S1*.*** Tumor-conditioned media contains soluble factors that promote the recruitment and generation of tumor-associated macrophages. Tumor-conditioned media contains inflammatory cytokines. Culture supernatants were harvested from 3 human cancer cell lines following 24 h of incubation in 0.2% FBS medium. A panel of cytokines and chemokines were quantified after being read on the Meso QuickPlex SQ 120. Data are presented as a heat map for expression of soluble factors in supernatants from each cell line. **Table S1**. List of PCR primers. Comprehensive list of PCR primers used in this manuscript. **Figure S2**. Additional functional characterization of in vitro generated TAM. M2-like macrophages and in vitro generated TAM were analyzed by real-time PCR for transcript expression of TNF-a (inflammatory mediator), VEGF (angiogenic factor), CSF-1R (colony- stimulating factor 1 receptor; overexpressed on TAM), and arginase (ARG1; a key factor in the suppressive function of TAM). (PPTX 140 kb)

## Data Availability

The datasets used and/or analyzed during the current study are available from the corresponding author on reasonable request.
