# Supplementary material for: Generation of monocyte-derived tumor-associated macrophages using tumor-conditioned media provides a novel method to study tumor-associated macrophages in vitro
Source: J Immunother Cancer. 2019 May 28;7:140. doi: 10.1186/s40425-019-0622-0 (PMC6540573; doi:10.1186/s40425-019-0622-0)
Supplement: Supplementary file 1 — Figure S1. Tumor-conditioned media contains soluble factors that promote the recruitment and generation of tumor-associated macrophages. Tumor-conditioned media contains inflammatory cytokines. Culture supernatants were harvested from 3 human cancer cell lines following 24 h of incubation in 0.2% FBS medium. A panel of cytokines and chemokines were quantified after being read on the Meso QuickPlex SQ 120. Data are presented as a heat map for expression of soluble factors in supernatants from each cell line. Table S1. List of PCR primers. Comprehensive list of PCR primers used in this manuscript. Figure S2. Additional functional characterization of in vitro generated TAM. M2-like macrophages and in vitro generated TAM were analyzed by real-time PCR for transcript expression of TNF-a (inflammatory mediator), VEGF (angiogenic factor), CSF-1R (colony- stimulating factor 1 receptor; overexpressed on TAM), and arginase (ARG1; a key factor in the suppressive function of TAM). (PPTX 140 kb) [file 40425_2019_622_MOESM1_ESM.pptx]

## Slide 1
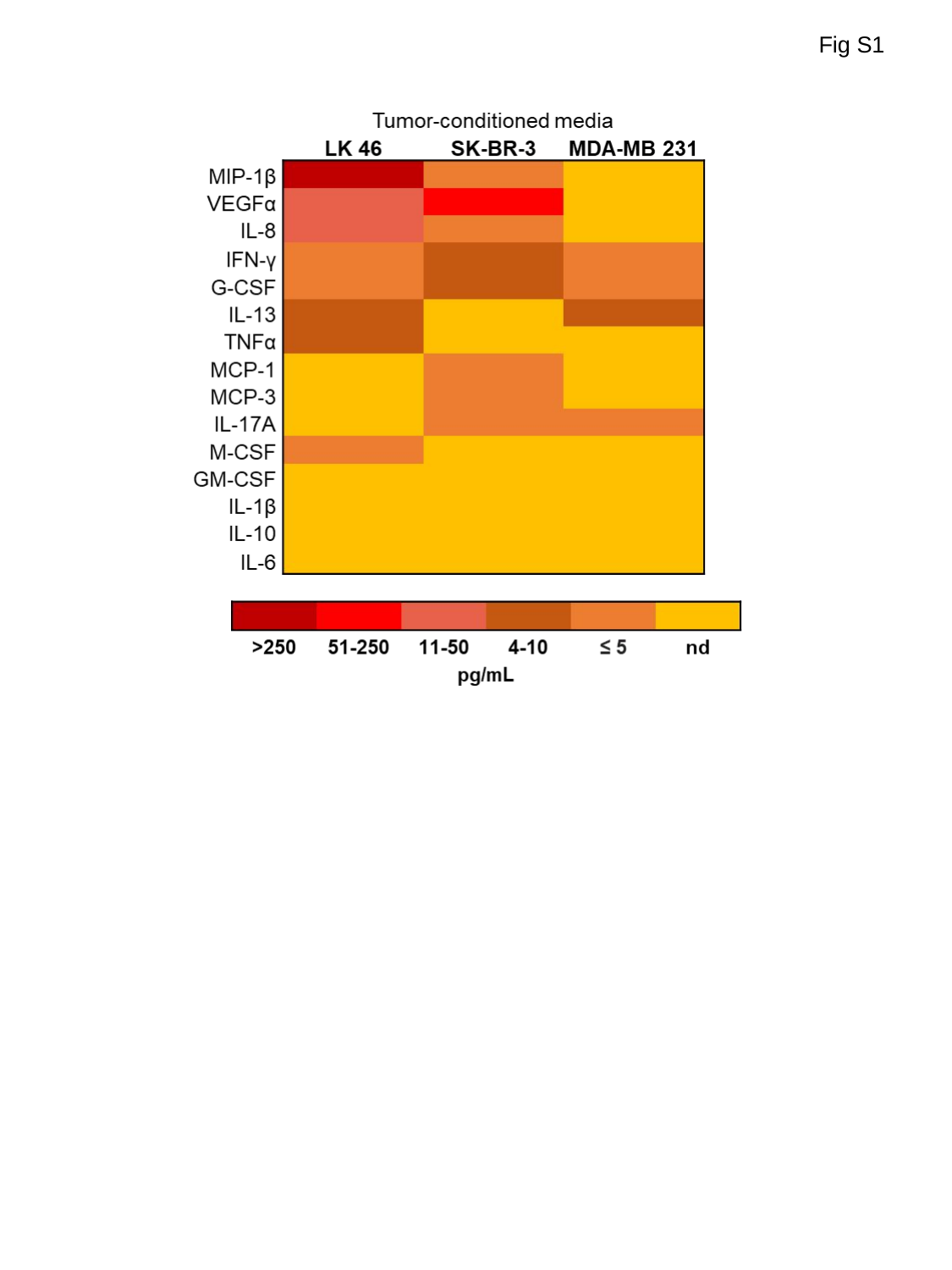

Fig S1

## Slide 2
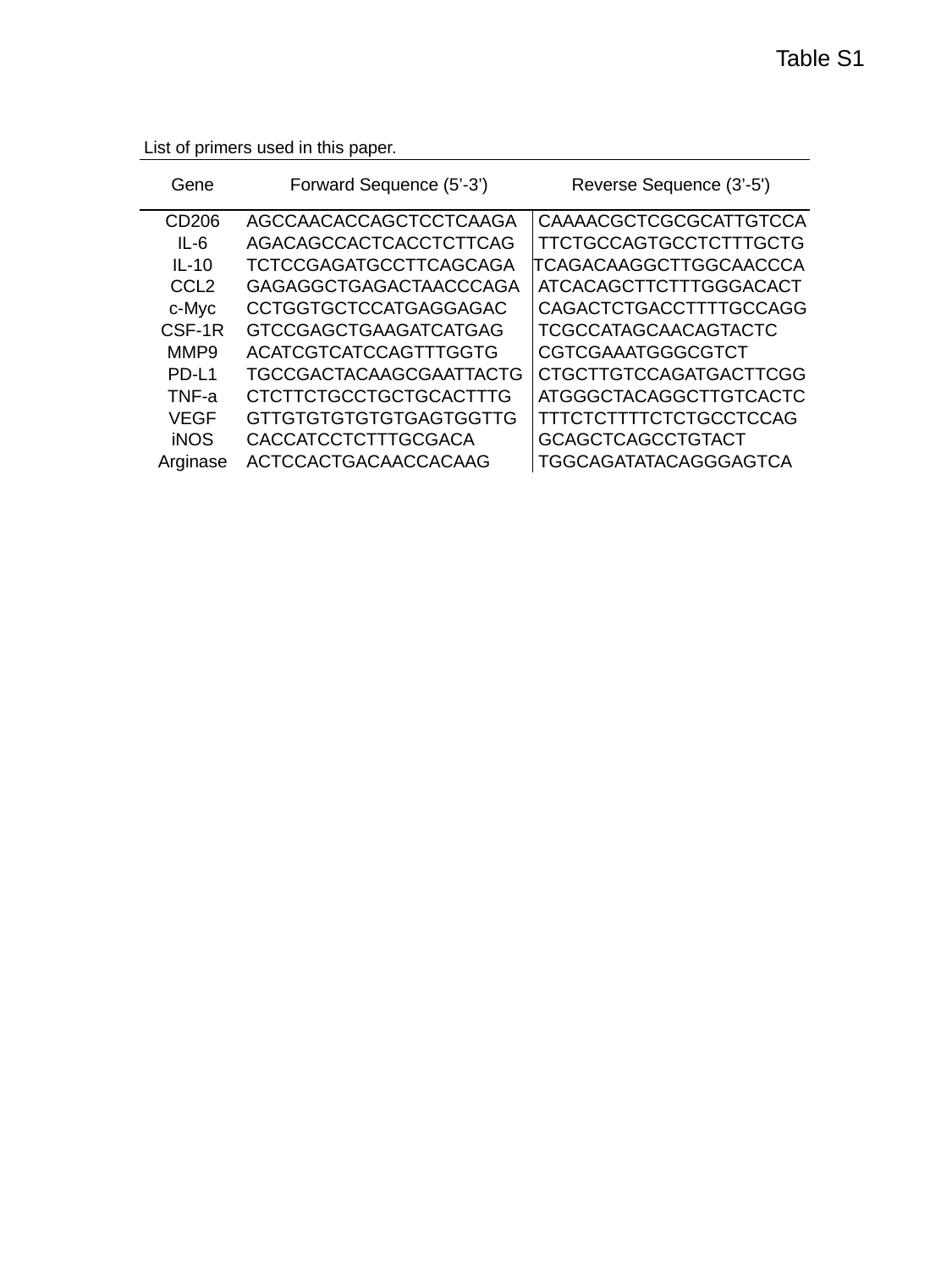

Table S1
List of primers used in this paper.
| Gene | Forward Sequence (5’-3’) | Reverse Sequence (3’-5') |
| --- | --- | --- |
| CD206 | AGCCAACACCAGCTCCTCAAGA | CAAAACGCTCGCGCATTGTCCA |
| IL-6 | AGACAGCCACTCACCTCTTCAG | TTCTGCCAGTGCCTCTTTGCTG |
| IL-10 | TCTCCGAGATGCCTTCAGCAGA | TCAGACAAGGCTTGGCAACCCA |
| CCL2 | GAGAGGCTGAGACTAACCCAGA | ATCACAGCTTCTTTGGGACACT |
| c-Myc | CCTGGTGCTCCATGAGGAGAC | CAGACTCTGACCTTTTGCCAGG |
| CSF-1R | GTCCGAGCTGAAGATCATGAG | TCGCCATAGCAACAGTACTC |
| MMP9 | ACATCGTCATCCAGTTTGGTG | CGTCGAAATGGGCGTCT |
| PD-L1 | TGCCGACTACAAGCGAATTACTG | CTGCTTGTCCAGATGACTTCGG |
| TNF-a | CTCTTCTGCCTGCTGCACTTTG | ATGGGCTACAGGCTTGTCACTC |
| VEGF | GTTGTGTGTGTGTGAGTGGTTG | TTTCTCTTTTCTCTGCCTCCAG |
| iNOS | CACCATCCTCTTTGCGACA | GCAGCTCAGCCTGTACT |
| Arginase | ACTCCACTGACAACCACAAG | TGGCAGATATACAGGGAGTCA |

## Slide 3
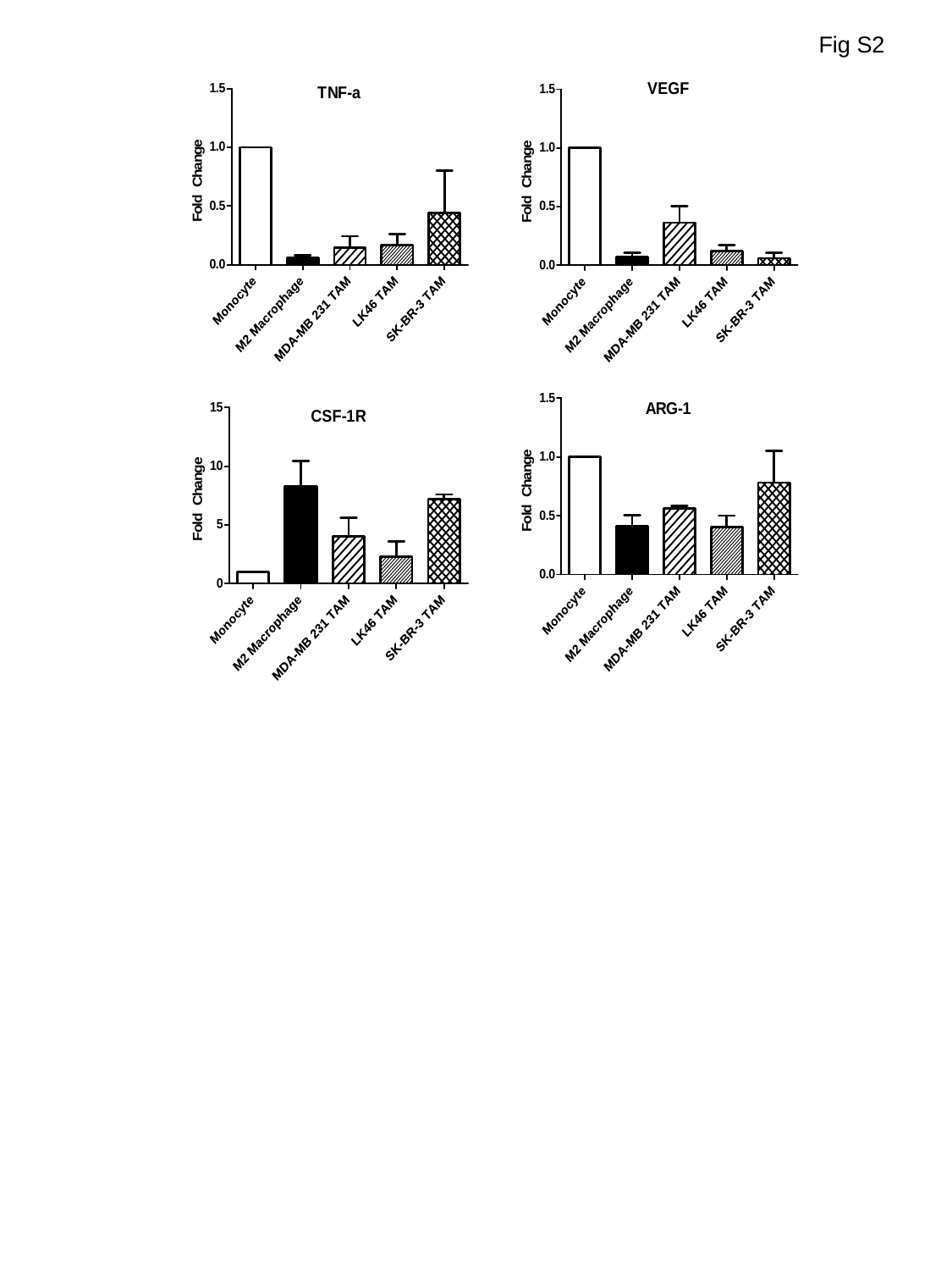

Fig S2
